# Role of MRP transporters in regulating antimicrobial drug inefficacy and oxidative stress-induced pathogenesis during HIV-1 and TB infections

**DOI:** 10.3389/fmicb.2015.00948

**Published:** 2015-09-17

**Authors:** Upal Roy, Paul Barber, Yuk-Ching Tse-Dinh, Elena V. Batrakova, Debasis Mondal, Madhavan Nair

**Affiliations:** ^1^Department of Immunology, Centre for Personalized Nanomedicine, Institute of NeuroImmune Pharmacology, Herbert Wertheim College of Medicine, Florida International UniversityMiami, FL, USA; ^2^Department of Chemistry and Biochemistry, Biomolecular Sciences Institute, Florida International UniversityMiami, FL, USA; ^3^Department of Molecular Pharmaceutics, Center for Nanotechnology in Drug Delivery, Eshelman School of Pharmacy, University of North Carolina at Chapel HillChapel Hill, NC, USA; ^4^Department of Pharmacology, Tulane University Health Sciences CenterNew Orleans, LA, USA

**Keywords:** MRP, HIV-1, TB, antimicrobials, oxidative stress, drug resistance, pathogenesis, therapeutic strategy

## Abstract

Multi-Drug Resistance Proteins (MRPs) are members of the ATP binding cassette (ABC) drug-efflux transporter superfamily. MRPs are known to regulate the efficacy of a broad range of anti-retroviral drugs (ARV) used in highly active antiretroviral therapy (HAART) and antibacterial agents used in *Tuberculus* Bacilli (TB) therapy. Due to their role in efflux of glutathione (GSH) conjugated drugs, MRPs can also regulate cellular oxidative stress, which may contribute to both HIV and/or TB pathogenesis. This review focuses on the characteristics, functional expression, and modulation of known members of the MRP family in HIV infected cells exposed to ARV drugs and discusses their known role in drug-inefficacy in HIV/TB-induced dysfunctions. Currently, nine members of the MRP family (MRP1-MRP9) have been identified, with MRP1 and MRP2 being the most extensively studied. Details of the other members of this family have not been known until recently, but differential expression has been documented in inflammatory tissues. Researchers have found that the distribution, function, and reactivity of members of MRP family vary in different types of lymphocytes and macrophages, and are differentially expressed at the basal and apical surfaces of both endothelial and epithelial cells. Therefore, the prime objective of this review is to delineate the role of MRP transporters in HAART and TB therapy and their potential in precipitating cellular dysfunctions manifested in these chronic infectious diseases. We also provide an overview of different available options and novel experimental strategies that are being utilized to overcome the drug resistance and disease pathogenesis mediated by these membrane transporters.

## The role of MRPs in decreasing therapeutic efficacy of HIV-1/TB drugs

Highly active antiretroviral therapy (HAART) has radically changed the clinical outcome of HIV through a substantial reduction of both mortality and morbidity. HAART is responsible for a 50% reduction in acquired immunodeficiency syndrome (AIDS) mortality rates. Furthermore, HAART has reduced maternal—infant transmission rates, decreased rates of opportunistic infections, and has led to a 40–50% reduction in the incidence of HIV associated dementia (Hayashi et al., 2006). HAART uses a combination of drugs that typically includes two different nucleoside reverse transcriptase inhibitors (NRTI), a non-nucleoside reverse transcriptase inhibitor (NNRTI) and a protease inhibitor (PI) or integrase inhibitor (INST). Both NRTIs and NNRTIs prevent the reverse transcription of HIV. INSTs inhibits the integration of HIV's DNA into the DNA of the infected cell. Lastly, PI's prevent the production of mature virions (Panel on Antiretroviral Guidelines for Adults and Adolescents, 2009). Despite the success of HAART, difficulties with this treatment strategy have emerged. Long-term side effects, sub-optimal drug potency, viral resistance, and the need for near-perfect adherence to therapy remain major barriers to achieve full and long-term viral suppression (Van Vaerenbergh, 2001). Viral resistance is a particularly challenging problem, as 30–50% of all individuals receiving HAART fail to respond to the drugs due to the development of drug resistance mechanisms. Thus, preventing possible escape of antiretroviral drugs by the viral population is essential for the efficient HAART. The viral infection depends on cellular factors at their site of action. In particular, two factors have been proposed: (i) the defective intracellular metabolism of nucleoside reverse transcriptase inhibitor (NRTI) in target cells; and (ii) the altered uptake and efflux of NRTI and protease inhibitor (PI) by cellular transporter molecules (Turriziani et al., 2003). Our previous study demonstrated that HIV-1 alone and HIV-1 in combination with a HAART drug, such as saquinavir, can significantly increase the drug efflux function of P-glycoprotein (P-gp) (Roy et al., 2012).

Approximately one third of the HIV-1 positive patients are also infected with TB (Ritchie et al., 2014). There is limited evidence that early intervention with HAART results in a better outcome in TB infection of dual infected patients (Yan et al., 2015). It has been suggested that HIV infection impairs host immune response to TB due to an over excitation of cytokine milieu in the co-infected host (Chetty et al., 2014). The overexpression of ABC transporter pump in HIV-1 and TB infection is associated with therapeutic failure. Since the discovery of ABC transporters, pharmacological ABC inhibitors have been explored as therapeutic targets with limited success (Sosnik, 2013). In this regard, a long acting treatment option is an absolute necessary to combat this disease. Some of the variability in patient response to anti-HIV and anti-TB therapeutics is due to the effect of single nucleotide polymorphisms (SNPs) on drug metabolism and drug transporter genes. It is known that SNPs in some genes that code for P450 and UGT enzymes play a major role in the altered expression and function of MRPs and P-gp; this is partly responsible for the inter-individual differences in efficacy and toxicity of anti-HIV and anti-TB therapeutics (Michaud et al., 2012). For instance, one study found that patients with naturally occurring mutation CYP2B6^*^6 of the CYP2B6 gene possessed higher plasma concentrations of the HAART drug, efavirenz (Tsuchiya et al., 2004).

**Figure 1:**
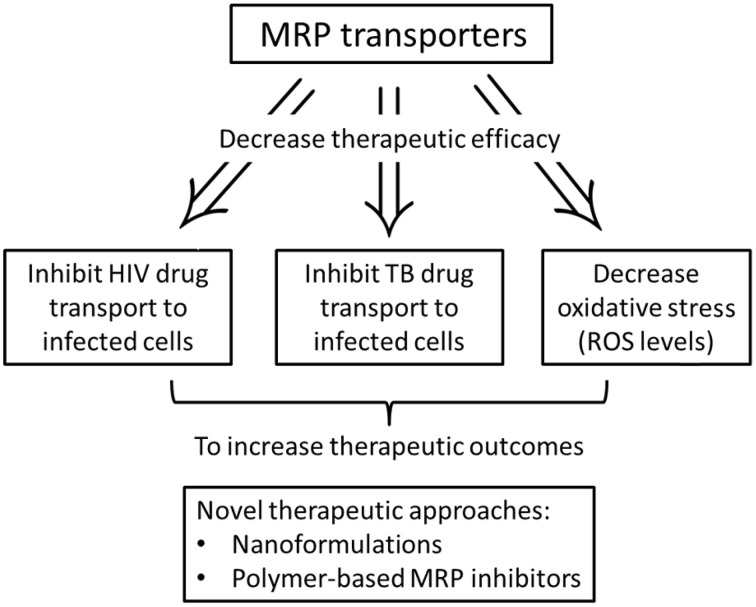
**Schematic representation of the role of MRP efflux transporters in HIV-1 and TB therapy**.

Multi-drug resistant proteins are part of the ABC superfamily of proteins that play an important role in the defense of cells against a wide range of xenobiotics. This family comprises a broad range of proteins found in organisms from bacteria to humans, and transport structurally diverse substances, such as ions, amino acids, sugars, peptides, and proteins across biological membranes. These drug efflux transporters have been implicated in the development of multi-drug resistance (MDR) (Figure 1). Cells selected for resistance to a cytotoxic drug may become cross resistant to a variety of drugs with different structures and cellular targets. MDR was thought to result exclusively from increase in P-gp activity encoded by the human MDR1 gene. However, researchers discovered that several cell lines selected for resistance did not show an increase in P-gp, yet became resistant to a range of natural product drugs (Zaman et al., 1994). In one of these non-P-gp MDR lines, the H69AR small cell lung carcinoma line found amplification and increased expression of a novel gene, the MRP (Cole et al., 1992). The overexpression of MRP has since been observed in several cell lines. Moreover, the subcellular location of MRP did not seem to be similar to that of a plasma membrane transporter, such as P-gp. This 190 KDa protein was found mainly in the endoplasmic reticulum rather than in the plasma membrane (Zaman et al., 1994). The discovery of MRP1 facilitated the discovery of eight more genes within the MRP family of which at least six (MRP2, MRP3, MRP4, MRP5, MRP6, and MRP8) are potentially involved in mediating drug resistance (Leslie et al., 2005). Noteworthy, breast cancer resistance protein (BCRP)—a transporter of the ABC superfamily—also acts to defend against toxins and xenobiotics by facilitating the excretion and limiting the absorption of potentially toxic substrate molecules. Known sites of BCRP expression include, the gut, bile canaliculi, placenta, testis, and brain (Natarajan et al., 2012).

Both P-gp and MRP1 are ATP dependent transporters. A common structural feature between P-gp and MRP1 is glycosylation (Boumendjel et al., 2005). P-gp transports structurally diverse drugs including, anthracyclines (doxorubicin, daunorubicin), vinca alkaloids (vinblastine, vincristine), paclitaxel, epipodophyllotoxins (etoposide, teniposide) and other compounds. MRP is remarkably similar to the drug transporting P-gp in its mode of action. Like P-gp, MRP1 (i) can promote resistance to various hydrophobic drugs, (ii) is located in the plasma membrane, as well as in the endoplasmic reticulum, (iii) can decrease drug accumulation in the cell through permeabilization of the plasma membrane, and (iv) can increase the efflux of drugs from cells (Zaman et al., 1994). Nevertheless, there are also important differences between P-gp and MRP1 with respect to the drugs that they transport and interact with. While P-gp drug efflux transporters can only target and transport hydrophobic drugs, MRP can transport hydrophilic molecules and even organic anions. MRP1 acts as a transporter of organic anions—particularly of glutathione conjugates—and can work in concert with the glutathione detoxification system (Váradi and Sarkadi, 2003). Studies involving MRPs are complicated by the presence of multiple biochemical pathways that can be utilized by the MRPs efflux system to extrude various compounds. In particular, glutathione S-transferases or GSH/GST detoxification system is known to act in concert with MRPs, as many MRPs substrates are GSH-dependent (Evers et al., 2000). These substrates (i.e., vinblastine, doxorubicin) are either co-transported with GSH, or first conjugated with GSH, and then transported as the GSH conjugate. MRPs can also transport neutral drugs conjugated with glutathione, glucuronide, or sulfate, and anticancer agents that are not metabolized to glutathione conjugates by co-transport with receded GSH. Usually GSH conjugation occurs either spontaneously or through catalysis by a glutathione S-transferase and may contribute to the MDR phenotype. However, it is not well understood how and why GSH doesn't interact with MRP1 (Boumendjel et al., 2005). The known substrates for BCRP include, methotrexate, mitoxantrone, and topoisomerase I inhibitors of topotecan and irinotecan (Kawabata et al., 2003).

MRP1 is ubiquitously expressed throughout the body, while MRP2 is most highly expressed in the liver, kidney and gut. The preferred substrates for MRP1 and MRP2 are organic anions, e.g., drugs conjugated with glutathione, glucuronate, or sulfate. Despite a similar substrate preference, MRP1 and MRP2 have distinct functions due to differences in their expression pattern. MRP1 is a major efflux pump that excretes a diverse range of endogenous substances and xenobiotics (He et al., 2011). MRP2 is involved in the excretion of bilirubin glucuronides into the bile and xenobiotics into the intestinal lumen. The next member of the family, MRP3, is concentrated in the liver and adrenal cortex and possesses a high structural similarity to MRP1 (58%). MRP3 has an affinity for glutathione and glucuronate conjugates, although its drug resistance capabilities are significantly less than either MRP1 or MRP2. In addition, MRP3 can transport monoanionic bile acids, which has led to speculation that MRP3 protein may help detoxify hepatocytes of bile acids (Kruh and Belinsky, 2003). MRP4 is predominately found in the kidney and lungs, whereas MRP5 is ubiquitously expressed. MRP4 and MRP5 act as organic anion transporters. Furthermore, both MRP4 and MRP5 are thought to mediate the urinary efflux of cAMP and cGMP (Maher et al., 2005). MRP4 was also found to play a significant role in the urinary excretion of furosemide and hydrochlorothiazide (Hasegawa et al., 2007). MRP6-MRP9 drug efflux transporters have been identified more recently and less is known about their function, localization and regulation. MRP6 is an organic anion transporter that is distributed most highly in the liver and kidney. While MRP6 does not play a role in drug resistance, it may be a constitutive “housekeeping” transporter of normal and abnormal hepatocytes (Madon et al., 2000). MRP7 is a lipophilic anion transporter primarily found in the heart, liver, skeletal muscle, and kidney. MRP7 has a substrate range similar to MRP1-MRP4 and is involved in phase III (cellular extrusion) of detoxification (Chen et al., 2003). MRP8 is an organic anion transporter that is highly expressed in the liver and has been associated with drug resistance to anti-cancer drugs. The precise function of MRP8 is currently unknown. Finally, MRP9 is known to be highly expressed in the testes and in breast tissue, but the preferred substrate and function of MRP9 is currently unknown (Yabuuchi et al., 2002).

## Evidence of MRP in efflux of Anti-HIV agents

HIV Protease Inhibitors (HPIs) are key components in HAART (O'Meara et al., 2001; Bachmeier et al., 2005). Given the impact of P-gp *in vivo* on HPIs pharmacology, it is important to assess, whether other drug transporters of the ABC family can also efficiently transport HPIs. For instance, HPIs are known to be substrates for MRP1 and MRP2. This may affect their pharmacological disposition and thus their therapeutic efficiency (Huisman et al., 2002). MRP1 is found throughout the human body and may have a role in resistance as it is also found in most tumors (Table 1). Without effective and specific MRP inhibitors, it's not possible to analyze the contribution of MRP1 to resistance by use of intervention studies, in which anticancer drugs transported by MRP1 are combined with an inhibitor of MRP1 (Borst et al., 2000). MRP2 is responsible for the transport of the majority of tested HPIs. This has essential implications for the pharmacological use of HPIs. In a rat model, MRP2 was shown to contribute to hepatobiliary, renal and direct intestinal excretion of its substrates, and to limit their oral bioavailability. It is highly likely that MRP2 reduces the plasma levels of HPIs through the same mechanism (Huisman et al., 2002). Of note, even within the same cell type, the resistance phenotype conferred by the expression of human MRP1 and mouse MRP1 differ substantially. In general, the resistance profiles of the various selected drugs were similar. It has been found that moderate to high level resistance occurs (depending on the cell line) to various drugs when MRP1 is over-expressed. While the resistance phenotype conferred by MRP1 expression may be influenced by the type of cell in which it is expressed, much of this variability is certainly the result of the complexity of cellular responses to drug selection (Hipfner et al., 1999).

**Table 1:** **Distribution, physiologic function, and substrate specificity of human multidrug resistance proteins**.

**MRPs**	**Location**	**Physiologic function**	**Substrate specificity**	**References**
MRP1	Ubiquitous	Efflux of a diverse range of endogenous substances and xenobiotics	Organic anions and steroid conjugates	He et al., 2011
MRP2	Liver, kidney, and gut	Excretion of bilirubin glucuronides into bile and xenobiotics into the intestinal lumen	Organic anions	He et al., 2011
MRP3	Liver, adrenal, pancreas, kidney, and gut	Acts as a protective mechanism when MRP2 is absent or nonfunctional. Also plays an important role in the enterohepatic circulation of endogenous compounds such as bile salts	Organic anions and monoanionic bile acids	Kruh and Belinsky, 2003
MRP4	Prostate, lung, muscle, pancreas, testis, ovary, bladder, and gallblader	Mediation of the extrusion of cAMP and cGMP in urine	Organic anions, cAMP, cGMP, and steroid conjugates	Maher et al., 2005
MRP5	Ubiquitous	Mediation of the extrusion of cAMP and cGMP in urine	Organic anions and cyclic nucleotides	Maher et al., 2005
MRP6	Liver and Kidney	Plays a vital constitutive housekeeping role in normal and abnormal hepatocytes.	Lipophilic anions	Madon et al., 2000
MRP7	Heart, liver, skeletal muscle, and kidney	Involved in phase III (cellular extrusion) of detoxification	Lipophilic anion	Chen et al., 2003
MRP8	Liver	Unknown	Cyclic nucleotides	Yabuuchi et al., 2002
MRP9	Breast tissue and testis	Unknown	Unknown	Yabuuchi et al., 2002

Most of the MRP1-mediated efflux is dependent on GSH. Thus, decreasing the intracellular level of GSH with L-buthionine-S, R-sulfoximine (BSO) and the inhibition of GSH synthesis reduces transport of Daunorubicin, Dosorubicin, Etoposide, and Vincristine in cells with over-expressed MRP1. These compounds are not known to conjugate with GSH. This implies that GSH is necessary for a possible co-transport mechanism or plays a role as a cofactor for substrate transport. It also seems that the transport efficiency is impacted by the hydrophobicity of the substrate. Hydrophobic substrates, such as derivatives of verapamil, enhance GSH transport of MRP1 more efficiently than hydrophilic derivatives. A direct interaction with drug binding sites is only one way that GSH affects MRP1. Drug transport may be also impacted by the increased ATP binding and ATPase activity caused by GSH. MRP2 and MRP3 also appear to be similar to MRP1 in the mechanism of GSH-mediated transport. GSH appears to be substrates for both MRP4 and MRP5 as well, but unlike other members of the MRP family, they also transport cyclic nucleotides cAMP and cGMP and even antiretroviral drugs 9-(2-phosphonylmethoxyethyl) adenine, which is independent of GSH (Ballatori et al., 2005). MRP1 is not an efficient transporter for sequinavir, ritonavir and indinavir (Huisman et al., 2002). Moreover, nelfinavir and amprenavir do not appear to be strong substrates for MRP1. The rank order of potency for MRP-related drug efflux transport was nelfinavir > ritonavir > sequinavir > amprenavir > indinavir (Bachmeier et al., 2005). Although interaction between the HIV-1 protease inhibitor, P-gp, and MRP drug efflux transporters has been reported, a quantitative assessment as to the extent of this interaction has not been performed, since this group of drugs is commonly utilized in combination with other HIV-1 protease inhibitor or various antiviral agents as part of a HAART regimen (Bachmeier et al., 2005).

## HAART induced oxidative stress

It has been shown that various conditions caused by different genetic or environmental insult may have one common molecular basis, namely, oxidative stress. Recent studies have indicated that HIV and TB infections are associated with oxidative stress (Tyagi et al., 2015). HIV increases the level of reactive oxygen species (ROS) within infected cells and the plasma/serum levels of lipid peroxidation products, malondialdehyde, and hydrogen peroxide. This is manifested in ongoing oxidative stress in HIV infected patients. The markers of oxidative stress previously stated can be detected in HIV infected T-cells, peripheral monocytes, and in the brain. Noteworthy, the brain is the main source of oxidative stress, as it is rich in polyunsaturated fatty acids, accumulates redox metal ions, consumes a large amount of inspired oxygen, is relatively low in antioxidants, and is composed largely of non-mitotic cells (Aksenova et al., 2005).

In HIV infection, oxidative stress caused by ROS may enhance viral replication by activating nuclear transcription factors, such as NF-κB, ultimately leading to viral gene expression. While the virus itself increases oxidative stress levels through replication, control of the virus with antiretroviral therapy leads to a similar increase in oxidative stress. HIV-infected subjects exhibited significantly higher levels of oxidative stress and DNA damage as measured by higher levels of modified lymphocyte DNA bases and lower activity levels of antioxidant enzymes than in HIV negative subjects (Lanzillotti and Tang, 2005). Free radicals can damage DNA through a number of mechanisms including direct alteration of base pairs resulting in miscoding. Some of these changes can be pre-mutagenic. Miscoding can also result in a decrease in critical proteins within neurons. DNA analyses of HIV infected lymphocytes demonstrated heightened hydroxylation of guanine and increased presence of thymidine/uridine residues when compared with lymphocytes from the control group (Valcour and Shiramizu, 2004). Recent reports continue to point to the mitochondria as the target for toxicity (Matarrese et al., 2005). The prevalence of these symptoms is worsened in individuals suffering from AIDS. The effect of oxidative stress as a consequence of mitochondrial toxicity may amplify some pathophysiological and phenotypic events during infection (Gil et al., 2005). Certain factors, such as immune activation due to HIV/co-infection and HAART increase the expression of HIV proteins, Tat, Nef, Vpr, and gp120. This induces oxidative stress, which can disrupt cell structure, alter blood brain barrier (BBB) defenses, and effect aberrant signaling and apoptosis (Perl and Banki, 2000; Valcour and Shiramizu, 2004). Oxidative stress plays a central role in the neurotoxicity caused by Tat and gp120. It has been confirmed that antioxidants can protect against the neural cell damage mediated by these virotoxins (Aksenova et al., 2005).

ROS produced by the host is critical for controlling TB infection. Unfortunately, very little is known about the dynamic response of TB to endogenous oxidative stress (Tyagi et al., 2015). Macrophage intracellular protein like NOD plays an important role in controlling inflammatory and ROS response in the cells. A proper homeostasis of ROS is essential for the persistence and survival of TB (Kumar et al., 2011). It is therefore suggested that anti-TB drug that induces ROS are at a greater risk of toxicity because of poor antioxidant mechanisms (Walubo et al., 1995).

## Role of MRPs in cellular oxidative stress

The potential importance of MRP1 in HIV infection relates to at least two crucial factors. The first is that HPIs and nucleoside analogs are both substrates for P-gp and MRP1. The second is that MRP1 plays a role in the defense against oxidative stress. In patients infected with HIV-1, increases in oxidative stress have been found to increase the transcription of HIV-1 by activating nuclear factor kB (NF-kB). The activation of MRP1 may play an important role in partial restoration under HAART (Lucia et al., 2005). MRP1 engenders resistance through the removal of drug conjugates from cells, which occurs indirectly through the transport of anionic phase II biotransformation products conjugated to GSH or other small molecules (Hipfner et al., 1999). The substrate with the greatest affinity for metabolites of exogenous molecules transported by MRP1 is GSH conjugated aflatoxin B_1._The *endo* and *exo* epo-oxides formed are detoxified by GST catalyzed conjugation to GSH. Following these formations, they are eliminated from the cell. Evidence of this process can be found in several transfection studies, which have shown that MRP1 and GST affect drug resistance through synergistic action (Hipfner et al., 1999). While MRP1 typically requires GSH in order to transport drugs out of cells, some drugs such as antimetabolites (MTX) can be removed from cells without GSH. (Létourneau et al., 2005). It was once thought that MRP1 simply transported glutathione S conjugates. However, we now know that the interplay between GSH and MRP1 is more complicated and only partly understood (Ballatori et al., 2005). GSH not only acts as a substrate of MRP1, but also has important roles in the overall transport mechanism. Furthermore, GSH plays a role in stimulating the transport of certain compounds by MRP1 (Létourneau et al., 2005).

## Accelerated drug resistance to HAART from drugs of abuse

**Figure 2:**
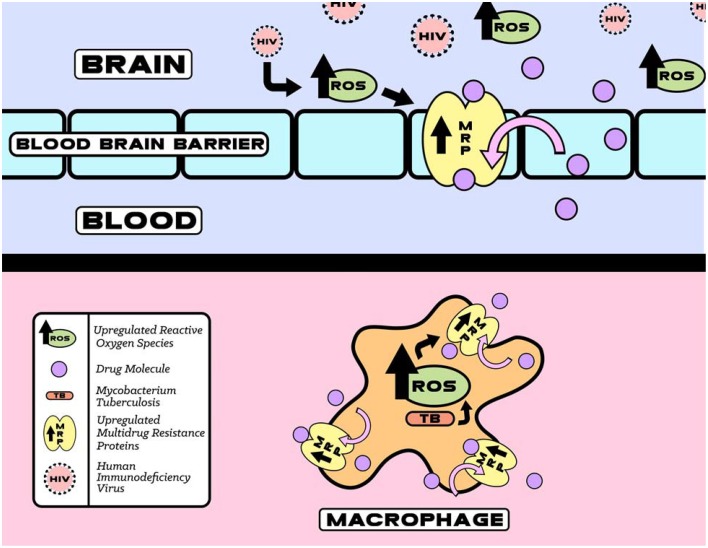
**HIV and TB induced upregulation of reactive oxygen species (ROS) that increases MRP function**. The increase in MRP mediated HIV and TB drugs efflux eventually decreases therapeutic efficacy.

Within a few days of infection HIV infected macrophages are extraverted through the BBB. Once inside the central nervous system (CNS), the neurotoxic effects of HIV are manifested indirectly through the release of viral proteins, such as gp120, from infected microglial cells. The increased expression of gp120 has been shown to cause neuronal damage both through the induction of oxidative stress and greater HIV penetration into the CNS due to alterations in the permeability of the BBB. Glial cell cultures treated with gp120 showed increased levels of ROS, apoptosis, lipid per oxidation, and a loss of dopaminergic neurons. In addition, these cells were found to have increased expression and activity of MRPs as shown in Figure 2 (Silverstein et al., 2012).

Cocaine and methamphetamine are known to accelerate HIV-associated neurocognitive disorders (HAND) through several factors. These drugs of abuse (DOA) enhance HIV-1 penetration into the CNS through (i) increased permeability of the BBB, (ii) increases in the transmigration of macrophages through the BBB (Zhang et al., 1998), and (iii) by exacerbating the neurotoxic effects of gp120 through decreased cell viability, an increased level of apoptotic cells, and increased expression of caspase-3, Bax, and ROS. Both of these DOA and gp120 were found to independently generate oxidative stress through overlapping signal transduction pathways (p38MAPK, NF-kB, etc.). When administered together, both cocaine and methamphetamine interact synergistically with gp120 to produce greater oxidative stress than is produced when administered separately (Silverstein et al., 2012).

HIV-infected individuals who suffer from drug addiction also experience accelerated resistance to HAART drugs (Silverstein et al., 2012). It is known that astrocytes undergoing oxidative stress rapidly release glutathione disulfide via MRP1 transporters (Hirrlinger et al., 2001). MRP1 can efflux all of the constituents of HAART from intracellular compartments (Eilers et al., 2008). Thus, we suspect that the additional oxidative stress placed on the brain through the abuse of cocaine/methamphetamine increases MRP1 expression at the BBB, thereby, accelerating resistance to HAART drugs.

## Role of MRPs in TB infection and therapy

TB is a major cause of morbidity and mortality for individuals infected with HIV. The complications from drug-drug interactions for concomitmant therapy of both conditions have been reviewed (Gengiah et al., 2011; Regazzi et al., 2014; Semvua et al., 2015). The first-line TB drug rifampicin (RIF) is a potent inducer of hepatic cytochrome CYP450 system, thus increasing the rate of metabolism of antiretroviral drugs in the liver to result in subtherapeutic antiretroviral drug concentrations (Piscitelli and Gallicano, 2001; Semvua et al., 2015). Protease inhibitors are known to be substrates of CYP3A4 and therefore could not be coadministered with RIF (Gengiah et al., 2011). RIF also increases the metabolism of integrase inhibitors by inducing the uridine diphosphate glucuoronosyltransferase (UGT) 1A1 enzyme (Wenning et al., 2009; Regazzi et al., 2014). Altered drug efflux from drug-drug interaction can also play a significant role in drug-drug interactions in treatment of patients coinfected with TB and HIV.

**Figure 3:**
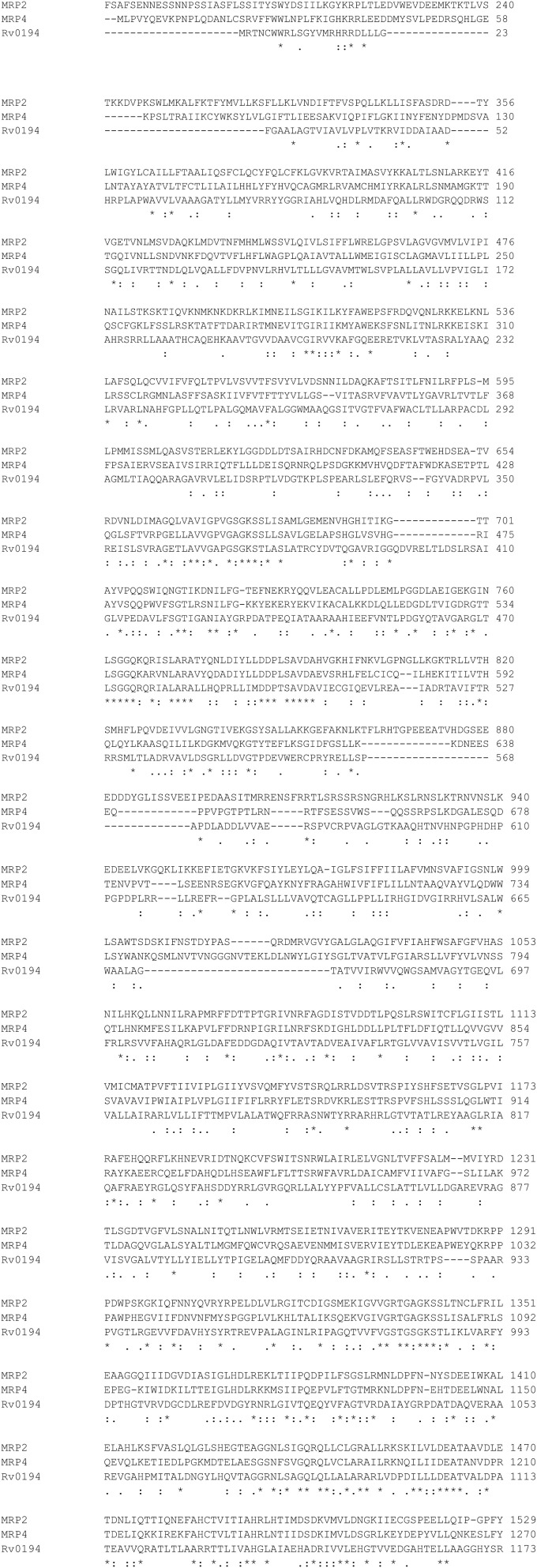
**Sequence Alignment of MRP2, MRP4, Rv0194 with ClustalW2**. http://www.ebi.ac.uk/Tools/msa/clustalw2/.

There is a large number of putative efflux pumps (EP) encoded in the *Mycobacterium tuberculosis* genome belonging to multiple major transporter families including the ABC-type transporters that are homologous to human MRPs (Black et al., 2014). DrrA (Rv2936) gene has been associated with efflux of multiple drugs including RIF, isoniazid (INH), and ethambutol (EMB) (Black et al., 2014; Li et al., 2015). Overexpression of ABC EP system Rv1456c-Rv1457c-Rv1458c was found in clinical isolates that are resistant to at least one of the four first-line drugs RIF, EMB, INH, and streptomycin (STR) (Hao et al., 2011). Increased expression of ABC transporters Rv1217c-Rv1218c in MDR-TB has been correlated with higher minimum inhibition concentrations (MICs) for RIF, and the overexpression of Rv1218c was correlated with higher MICs for INH (Wang et al., 2013). Another ABC transporter found in *M. tuberculosis*, Rv0194, has also been associated with resistance to a variety of antibiotics including STR (Danilchanka et al., 2008). Sequence alignment of Rv0194 with human MRP2, MRP4 is shown in Figure 3.

If human MRPs can also act as efflux pumps for the TB drugs, the increased expression of MRPs in macrophages would decrease the intracellular levels of the TB drugs (Figures 1, 2). This would aid the evolution of TB drug tolerance (Szumowski et al., 2013). The expression levels of MRP in the peripheral mononuclear cells of MDR-TB patients were found to be approx. three-fold higher than non-TB control group and TB control group (Lian et al., 2011). This may be responsible for the multi-drug resistance in MDR-TB patients. A study of *Listeria monocytogenes* has shown that a bacterial efflux pump may cooperate with macrophage MRP to reduce the antibacterial activity of the common substrate ciprofloxacin (Lismond et al., 2008). Moxifloxacin is a second line TB drug currently used in novel regimens designed to decrease length of therapy (Dawson et al., 2015). Unlike the more commonly prescribed fluoroquinolone, ciprofloxacin, moxifloxacin accumulation is not affected by overexpression of MRP4 in macrophages (Michot et al., 2006). Prolonged exposure to moxifloxacin can nevertheless result in the overexpression of macrophage transporters including P-gp, MRP2, and MRP4 resulting in the drug-drug interaction (Vallet et al., 2011).

## Novel approaches to overcome multi-drug resistance to HIV and TB therapy

Since the emergence of HIV, decades of research has focused on the treatment of AIDS to combat the virus. These treatments have transformed the outcome of AIDS from a subacute illness with near certain death to a chronic disease with hope for long term survival for many patients. Present research has centered in on the long term survival of HIV in the body, the side effects of the treatment, and long term consequences of infection. Several studies related to the MRP inhibition—mainly MRP1 and MRP2—have found that even short term inhibition results in unacceptable levels of toxicity (Higgins, 2007).

One clinical trial found that selenium supplementation in HIV positive subjects reduced hospitalization rates and declines in CD4+ cell counts as compared to subjects who were not given the supplement. While selenium is an integral part of glutathione peroxidase—a major protective enzyme against oxidative stress—it also plays an important role in immunologic function. During viral infection, it appears as though the Th1 series of cytokines is necessary for an appropriate cellular defense. These include Interleukin 2 (IL 2) and Interferon γ (IFN-γ). Several studies have found that as HIV infection progresses, IL-2 and IFN-γ levels decline, while Th2 cytokines, specifically IL-4, IL-6, and IL-10 increase. In *in vitro* studies, selenium supplementation has been shown to up-regulate IL-2 and thus increase the activation, proliferation, and differentiation of T-helper cells (Lanzillotti and Tang, 2005).

Usually natural product derived drugs enter cells by passive diffusion. Oxidation and/or conjugation can inactivate these amphipathic drugs. Conjugation alone is not sufficient to get rid of the drugs. The conjugated drugs are no longer hydrophilic due to the attachment of GSH, and therefore, they are unable to leave the cell via passive diffusion. As the drug continues to enter the cell, and get conjugated to the GSH, it will accumulate to excessive concentration unless get exported by a specific export pump, GS-X pump (Borst et al., 2000). The profound drug-drug interaction between immunosuppressive drugs and a protease inhibitor have been observed. Without the use of protease inhibitors, HAART with nucleoside or non-nucleoside reverse transcriptase inhibitors has been shown to produce less significant drug-drug interactions. Thus, it is vital to understand these potential pharmacodynamic drug-drug interactions in order to avoid drug toxicity and lack of efficacy (Izzedine et al., 2004). The HAART-induced mitochondrial toxicity has indicated particularly dideoxynucleotide reverse transcriptase inhibitor partially through inhibition of mitochondrial DNA polymerase gamma, and this mitochondrial damage is associated with acute and chronic viral infection (Valcour and Shiramizu, 2004). It has been established that changes in the ATP production or mitochondrial metabolism of cancer cells impacts drug resistance and redox balance (Lucia et al., 2005).

## Nanomedical applications toward multi-drug resistance to HIV and TB therapy

For several widely used antiretrovirals, for example efavirenz, the drug resistance is engendered through poor bioavailability at anatomical viral reservoir sites. One of these sites, the CNS, is vital to HIV persistence, and acts as a major barrier to successful HIV eradication. Following antiretroviral therapy, HIV persists in the long-lived cells of the CNS as a latent infection. At sub-therapeutic antiretroviral levels, the CNS reservoir provides opportunity for the virus to evolve and enhance its fitness (Gomes et al., 2014). Most antiretroviral drugs cannot reach these sites in therapeutic doses, because they are either unable to penetrate the CNS, or they are excluded by efflux transporters in the BBB. In addition, the long-term presence of HIV in the CNS has been associated with neurocognitive and motor disorders (Gomes et al., 2014). Rates of HIV associated neurocognitive impairment will likely rise in the coming years as anti-HIV therapies continue to extend the lifespan of patients. Eliminating CNS reservoirs of HIV will greatly increase the quality of life and lifespan of seropositive individuals. Engineered nanoparticles may provide the ability to bypass the BBB and reach these HIV reservoir sites. Previous studies have indicated that polymeric nanoparticles loaded with HAART drugs can cross BBB and reach the CNS reservoir at therapeutic levels in HIV-1 infected humanized mouse and macaque models (Dash et al., 2012; Roy et al., 2012). It also been established that the sustained drug release provided by these nanoformulations are more effective in controlling viral replication than the unformulated drugs that are currently available (Gautam et al., 2013). Therapeutic drugs conjugated to nanodiamond may be able to overcome the restricted access of the BBB by stealthily bypassing efflux transporters. These nanoparticles should have a total size ranging from 4 to 100 nanometers in order to avoid efflux into the extracellular membrane. The nanodiamond core will also increase drug stability and prolong drug circulation time (Jarre et al., 2014). Coating the nanoparticles with a surface stabilizer may be of interest in achieving increased brain drug levels. In particular, the addition of polysorbate 80—a nonionic surfactant—may significantly enhance brain delivery. One study showed a 20-fold increase in brain endothelial cell uptake of polysorbate 80-coated nanoparticles compared to the uncoated nanoparticles. Increased uptake of the coated nanoparticles is thought to result from polysorbate 80's adsorption of different apolipoproteins, thereby mimicking lipoproteins on their receptor-mediated transcytosis pathway into the CNS (Ramge et al., 2000).

Polymeric micelles were also suggested for improved transport of drugs across the BBB and to drug-resistant cells. These carriers consist of amphiphilic polymers that spontaneously form nanosized aggregates when the individual polymer chains (“unimers”) are directly dissolved in aqueous solution (Kabanov et al., 1995) above a threshold concentration (critical micelle concentration or CMC) and solution temperature (critical micelle temperature or CMT). Polymeric micelles can be utilized for the delivery of hydrophobic and amphiphilic drugs. They offer several benefits as nanocarriers, including, improved drug solubility, protection of drugs from degradation, and increased drug exposure time to specific tissues. Polymeric micelles have been evaluated in several pharmaceutical applications as drug and gene delivery systems (Kabanov et al., 1995; Löbenberg et al., 1998a,b; Bronich et al., 2000; Kabanov and Alakhov, 2002; McGirt et al., 2004; Shah and Amiji, 2006; Kabanov and Batrakova, 2008). Recently, new polymer micelles-based nanosystems were developed that utilize cross-linked ionic cores. These novel polymer micelles allow for the encapsulation of charged therapeutic or diagnostic molecules and resist dissociation of the micelle upon dilution (Bronich et al., 2000).

In addition, selected polymer nanomaterials can be used for biological response modifiers. One example of these polymer nanomaterials is Pluronics—triblock copolymers consisting of poly(ethylene oxide) (PEO) and poly(propylene oxide) (PPO), (PEO-b-PPO-b-PEO), which has been found to cause various functional alterations in cells (Batrakova and Kabanov, 2008). It was demonstrated that a formulation of different low molecular weight drugs, including antiretroviral drugs, into Pluronics resulted in the improved drug transport across the BBB. The mechanism of these effects is rather complex. Pluronics, in contrast to the majority of low molecular mass inhibitors of drug efflux transporters that are designed to interact specifically with the transport system protein, are known to perform a wide range of activities. They inhibit P-gp and MRP drug efflux proteins (Batrakova and Kabanov, 2008) through interaction with MDR cell membranes by decreasing the membrane microviscosity and inhibiting the P-gp and MRP ATPase activity (Batrakova et al., 2001, 2003). They are also known to inhibit respiratory chain complexes in the mitochondria of MDR cells. This diminishes ATP and depletes MDR cells of energy (Batrakova et al., 2001). Furthermore, Pluronics causes a sharp reduction in GSH and GST activity, thereby inhibiting glutathione/glutathione *S*-transferase (GSH/GST) resistant mechanisms (Batrakova et al., 2003). In addition, they may improve the bioavailability of drugs within resistant cells by attenuating their sequestration in acidic vesicles (Venne et al., 1996). Lastly, Pluronics have been found to reduce membrane potential in the mitochondria of MDR cells. This causes an overall increase in pro-apoptotic signaling and decrease in anti-apoptotic cellular defense of MDR cells via increase in cytochrome C release (Minko et al., 2005). Surprisingly, Pluronics appear to be selective with respect to the MDR cell phenotype despite their rather simple structure and lack of precise spatial arrangement of pharmacophoric groups (Batrakova et al., 2001). This is most noticeably seen in ATP depletion by Pluronics, which correlates with the level of drug efflux transporters expression in the resistant cells (Batrakova et al., 2003). Of course, the more complete understanding of these issues is necessary for the future development of drug delivery system based on polymer surfactants. Nevertheless, this strategy has a potential in developing novel modalities for delivery of various drug to the brain, including HPIs to eradicate HIV virus in the brain. Recent development on long acting nanoparticle against both HIV-1 and TB suggested that gallium-based anti-HIV and TB nanodrug can control HIV and TB replication in dual infected human macrophages. However, whether it can bypass MRP efflux has not been tested so far (Narayanasamy et al., 2015).

## Conclusion

The MRP family forms the major efflux system involved in efflux transport of anti-HIV drug out of cells. The expression profile of certain MRPs in the cell membrane has not been well characterized. While the presence of MRP1-MRP9 has been established in different cell lines (Borst et al., 2000), the function and activity of most members remains speculative. Understanding the role of MRP family is crucial to establishing effective HAART, as PI and NRTIs are good substrates for MRPs. Published reports suggest that oxidative stress, alteration of glutathione metabolism and activation of the MAP kinase can be involved in MRP expression (Guan et al., 2004; Hayashi et al., 2006). Hayashi et al., 2006 also suggested that HIV transcription regulator, Tat protein can specifically up-regulate MRP1 which may contribute to the development of resistance to HAART. Up-regulation of MRP1 also has the potential of increasing the efflux of anti-TB drugs. It is important to focus to research on signaling cascade that triggers the altered expression of MRPs. The fact that MRPs are present in different forms in different cell membranes makes studying them difficult. Inhibiting specific MRPs—even for short periods of time—is not tolerated well in patients. Increased understanding in this field will enable researchers to design more focused methods to inhibit HIV replication.

### Conflict of interest statement

The authors declare that the research was conducted in the absence of any commercial or financial relationships that could be construed as a potential conflict of interest.
